# 654 Women with Intentional Flame Burns: Findings from the World Health Organisation’s Global Burn Registry

**DOI:** 10.1093/jbcr/iraf019.283

**Published:** 2025-04-01

**Authors:** Claudia Malic, Yvonne Singer, Emily Bebbington, Raquel Pan

**Affiliations:** Ottawa Children’s Hospital of Eastern Ottawa; Griffith University; Bangor University; Federal University of Triângulo Mineiro

## Abstract

**Introduction:**

The World Health Organization (WHO) estimates one in three women worldwide have experienced physical or sexual violence by an intimate partner. Very little is known about the burden of burn violence against women. Emerging evidence suggests women who experience burn violence are young, sustain severe injuries and have high mortality. Individual burn centers around the world have contributed data to the publicly accessible WHO’s Global Burn Registry (GBR) since 2017, that includes information about household intentional flame burns injuries. Despite the inability to determine if harm was self-inflicted or caused by others, we have quantified the data.

The aim of this study was to describe the frequency, demographic characteristics, injuries, and outcomes of women with household intentional flame burns admitted to burn centers that contributed data to the GBR.

**Methods:**

We extracted all data from the GBR for women (≥ 18 years) admitted to GBR contributing burn centers with household intentional flame burn injuries. Simple statistical analysis was performed for demographics, injury characteristics, and in-hospital outcomes.

**Results:**

Between 2017 and 2022, 208 women with intentional flame burn injuries were registered in the GBR, 77.9% were from LMICs, predominantly India (58.1%). Most women were aged between 20-29 years old (35.7%) or 30-39 years old (29.0%) and the majority were illiterate (77.1%). Their burn injuries were extensive, 68.3% has burns >50%TBSA, and 27.6% had burns >90%TBSA. Only 8.1% has burns < 20%TBSA. The majority of women had inhalation injury (71.9%). Most injuries involved kerosene (67.1%). Overall, 145 women died (69%) and 8.6% discharged against medical advice.

**Conclusions:**

This descriptive study contributes to a growing body of international literature demonstrating the confronting nature and devastating outcomes for women who experience intentional burn injuries. Our findings are an extreme underestimation of the true number of women with burns caused from violence admitted to burn centers, as the GBR collects data about people with burn injuries from a small fraction of burn centers globally, and women do not always disclose violence. Our findings also likely have considerable selection bias, as we analysed only a subsample of GBR injury intent data that was publicly available. We have not accounted for women who sustained intentional flame burns outside the home or other types of burns. Moreover, no new cases have been entered into GBR since 2022.

**Applicability of Research to Practice:**

The need to re-establish data collection, particularly in LMICs must be prioritized to close the gap in knowledge about the burden of burn injury, as well as burn violence.

**Funding for the Study:**

N/A

